# Neural substrates of similarity and rule-based strategies in judgment

**DOI:** 10.3389/fnhum.2014.00809

**Published:** 2014-10-15

**Authors:** Bettina von Helversen, Linnea Karlsson, Björn Rasch, Jörg Rieskamp

**Affiliations:** ^1^Department of Psychology, Center for Economic Psychology, University of BaselBasel, Switzerland; ^2^Department of Integrative Medical Biology and Umeå Center for Functional Brain Imaging, Umeå UniversityUmeå, Sweden; ^3^Department of Psychology, University of FribourgFribourg, Switzerland

**Keywords:** judgment and decision making, fMRI, exemplar model, cognitive strategies, cognitive modeling, multi-attribute decision making

## Abstract

Making accurate judgments is a core human competence and a prerequisite for success in many areas of life. Plenty of evidence exists that people can employ different judgment strategies to solve identical judgment problems. In categorization, it has been demonstrated that similarity-based and rule-based strategies are associated with activity in different brain regions. Building on this research, the present work tests whether solving two identical judgment problems recruits different neural substrates depending on people's judgment strategies. Combining cognitive modeling of judgment strategies at the behavioral level with functional magnetic resonance imaging (fMRI), we compare brain activity when using two archetypal judgment strategies: a similarity-based exemplar strategy and a rule-based heuristic strategy. Using an exemplar-based strategy should recruit areas involved in long-term memory processes to a larger extent than a heuristic strategy. In contrast, using a heuristic strategy should recruit areas involved in the application of rules to a larger extent than an exemplar-based strategy. Largely consistent with our hypotheses, we found that using an exemplar-based strategy led to relatively higher BOLD activity in the anterior prefrontal and inferior parietal cortex, presumably related to retrieval and selective attention processes. In contrast, using a heuristic strategy led to relatively higher activity in areas in the dorsolateral prefrontal and the temporal-parietal cortex associated with cognitive control and information integration. Thus, even when people solve identical judgment problems, different neural substrates can be recruited depending on the judgment strategy involved.

## Introduction

Making accurate judgments is an important human competence. Doctors and judges routinely make judgments that may decide whether someone lives or dies. Judgments usually are made on the basis of several pieces of information or cues. For instance, a doctor may set the medication dosage for a patient by considering the severity of the symptoms and the patient's medical history. Naturally, the neural substrates underlying judgments have raised the interest of numerous researchers (e.g., Greene and Haidt, 2002; Paulus and Frank, 2003; Heekeren et al., 2005; Moll et al., 2005; Knutson et al., 2007; Hare et al., 2009; Pine et al., 2009). However, so far, little attention has been paid to the fact that identical judgment problems can be solved by different judgment strategies. This implies that the variety in neural substrates underlying human judgments could result from different judgment strategies. The majority of imaging studies have largely ignored people's strategies or focused on a single strategy (Zysset et al., 2006; Kahnt et al., 2011; Khader et al., 2011). This is surprising because a plethora of research in cognitive psychology suggests that people frequently adopt a variety of different strategies in cognitive tasks such as judgment, decision making or problem solving (e.g., Gigerenzer and Goldstein, 1996; Ashby et al., 1998; Lemaire, 2002; Juslin et al., 2008; Rieskamp and Hoffrage, 2008; von Helversen and Rieskamp, 2008). In this vein, a distinction between similarity-based and rule-based strategies has been drawn in research on judgment and this is supported by a large amount of behavioral evidence (see e.g., Karlsson et al., 2007; Juslin et al., 2008; Hoffmann et al., 2013; von Helversen et al., 2013). People's use of different judgment strategies should be reflected at the neural level. Indeed, in categorization research a related distinction between similarity-based and rule-based strategies has led to research suggesting that these categorization strategies largely rely on different brain regions (for reviews see Nomura and Reber, 2008; Smith and Grossman, 2008). In the current work we will investigate the neural underpinnings of similarity-based and rule-based judgment strategies.

### Similarity- and rule-based strategies in judgment and categorization

Overall, categorization and judgment tasks share many features as both involve the evaluation of an object based on its attributes or characteristics. However, they differ in the type of response that is required. Whereas categorization involves a dichotomous decision such as deciding whether a patient needs medication or not, judgments require a more fine-grained response such as estimating the exact dosage that is prescribed. Despite these differences, it has been suggested that people rely on similarity-based and rule-based strategies in both tasks. Similarity-based strategies in judgment and categorization follow the assumption that category decisions or judgments are made based on the similarity of the current object under evaluation to previously encountered instances that are retrieved from memory (Medin and Schaffer, 1978; Juslin et al., 2003). For instance, a doctor facing the problem how to set the medication dosage for a patient could think back to similar patients and the medication dosage they required. In contrast, rule-based strategies usually follow the assumption that people make judgments or decisions by applying a previously abstracted rule that defines how the characteristics or features of the object under evaluation relate to the decision criterion. Following this strategy the doctor would, for instance, assign a high dosage if the patient had severe symptoms, but did not fall into a risk group.

In comparison to the similarity-based approach, rule-based strategies do not necessarily require the retrieval of previously encountered instances, but require keeping the rule active in working memory (e.g., Bruner et al., 1956; Hahn and Chater, 1998; Juslin et al., 2003; Hoffmann et al., 2013, in press). In general, this suggests that similarity-based strategies should lead to relatively higher activation compared to rule-based strategies in the *left inferior* and *anterior prefrontal cortex* (aPFC) and *the inferior parietal cortex* (IPC)—areas that past research has strongly connected to memory retrieval and comparison processes (e.g., Badre and Wagner, 2007; Martin, 2007; Spaniol et al., 2009; Kim, 2011; Grossman et al., 2013). In contrast, using a rule-based judgment strategy should lead to relatively higher activity in the *dorsolateral prefrontal cortex* (dlPFC) and the *anterior cingulate cortex*, areas that have been strongly connected to cognitive control and rule use (e.g., Grossman et al., 2002; Bunge, 2004; Heekeren et al., 2004).

Largely in line with these hypotheses, past research in categorization has demonstrated that relying on rule-based strategies leads to relatively higher activation in the dlPFC and the anterior cingulate cortex than similarity-based categorizations. However, frequently a higher activation in the posterior parietal cortex (PPC)—an area associated with selective attention—has also been found (Patalano et al., 2001; Grossman et al., 2002; Koenig et al., 2005). Consequently, if using similarity- and rule-based strategies in judgment involves cognitive demands similar to their counterparts in categorization, one would also expect a higher activation in the PPC for rule-based judgment strategies.

Although similarity- and rule-based judgment strategies share a conceptual basis with the respective strategies in categorization, they also differ in important aspects. For instance, rule-based strategies in categorization and judgment may differ in the cognitive demands they pose: Research in categorization has mostly compared rule-based strategies that rely on a single dimension with similarity-based strategies that consider similarity on several dimensions (Erickson and Kruschke, 1998; Grossman et al., 2002). For instance, when deciding whether an unknown bug is toxic one could follow a single dimensional rule that bugs in warning colors such as red or yellow tend to be dangerous and ignore other characteristics such as the bug's size. In contrast a similarity-based strategy would rely on the overall similarity of the bug in question to bugs one knows to be dangerous, taking into account other features such as size or the type of wings besides the coloring. In contrast, in judgment tasks usually both rule- and similarity-based strategies are assumed to integrate information on several dimensions (Juslin et al., 2008; von Helversen and Rieskamp, 2008). For making a fine-grained judgment it is usually necessary to consider several characteristics of the object. For instance, a rule-based judgment strategy could identify a bug as medium toxic if about half of its characteristics are typical for toxic bugs. This difference between judgment and categorization strategies could affect which brain areas are activated. To the degree that the higher activation in the PPC frequently found in rule-based categorization processes reflects selective attention to only one or two characteristics (e.g., Grossman et al., 2002), it is possible that a different pattern of activation would be found in a judgment task.

The goal of the present research was twofold. First, we aimed at investigating whether similar to categorization tasks an identical judgment task recruits different brain areas depending on whether a rule-based or a similarity-based judgment strategy was followed. Secondly, we wanted to examine whether similarity-based and rule-based judgment strategies have neural correlates similar to categorization strategies. Specifically, we investigated whether rule-based judgment strategies lead to a higher activation in the dlPFC and the PPC, and similarity-based strategies result in higher activation in the aPFC and IPC.

To investigate these questions, we conducted an experimental study where we compared the neural activations when relying on a similarity-based judgment strategy and a rule-based judgment strategy. In the experiment participants performed two judgment tasks. These two tasks were selected from three different judgment scenarios. For instance, in one medical scenario, participants had to estimate the medication dosage for a patient that could range between 200 and 300 mg based on the patient's symptoms (the cues). The patients differed on six cue (symptom) dimensions that could be used to make the judgment. For example, the patient's blood pressure could be high or low, the worst time period could be in the morning or the evening, and the virus could be type A or B. In another employment scenario, the task was to evaluate the suitability of a job applicant for an IT position based on the applicants' knowledge in different fields such as foreign language skills or special qualifications. In the last biological scenario, the task was to estimate the amount of toxin in a bug's saliva based on the bug's appearance. Each participant performed one judgment task with each strategy, each with a different scenario. The third scenario was used as a distractor task in the functional magnetic resonance imaging (fMRI) session.

Because we were interested in how participants apply rule- and similarity-based judgment strategies, we instructed participants to solve the judgment tasks using either a typical rule-based strategy—a judgment heuristic—or a typical similarity-based strategy: an exemplar-based strategy (von Helversen and Rieskamp, 2009a). The content of the two judgment tasks differed, but both tasks had the same underlying structure. In our subsequent analysis, we focused on comparing the cognitive processes of the two judgment tasks against each other.

## Materials and methods

### Participants

Twenty-three right-handed, healthy participants, with normal or corrected to normal vision, were recruited to take part in the experiment (17 women, mean age = 20.13, *SD* = 2.67). Participants reported no psychiatric or neurological disorder. Written informed consent was obtained from all participants after the study procedures had been explained. The Basel canton ethics committee approved all procedures in this study. We excluded five participants from the analyses: Three participants were excluded because of excessive head movement (>3 mm) and two participants because modeling their judgments suggested that they did not use the instructed strategies (see details in behavioral results).

### General procedure

To ensure that participants learnt to apply the two judgment strategies accurately, we conducted the study on two consecutive days. On the first day, participants learnt to solve the judgment tasks by using the two judgment strategies; on the second day, they solved trials from both judgment tasks switching between strategies while we recorded fMRI data.

### Judgment task

#### Stimuli

The participants' task was to make fictitious quantitative judgments of several items using a scale with 100 possible values. Each item was described by six binary cues and a criterion value (see Table 1). The task was to learn to estimate the correct criterion value of items given the items' cue values. In Table 1 cue values are denoted by “0” or “1,” where a value of “0” indicates a “negative” cue value and “1” a “positive” cue value. A positive cue value means that on average an item with a “1” for this cue will have a higher criterion value than an item with a “0” for this cue. Thus a positive cue value indicates a higher criterion value than a negative cue value. The criterion values for each item were determined by a multiplicative function of the cue values (for the function used, see von Helversen and Rieskamp, 2009a). To help participants to be able to switch between the two judgment strategies according to the instruction and to control for content specific effects, participants learnt each strategy using a different judgment task scenario. Overall, we used three different judgment scenarios that had the same underlying information structure but differed in content: (1) a medical task in which participants judged the medication dose for a patient based on the patient's symptoms (i.e., the cues) such as location of headache (front or back), blood pressure (high or low), color of mucus (yellow or green), or type of rash (reddening or itching), (2) an employment task in which participants rated the quality of a job applicant for an IT position based on the applicant's characteristics such as foreign language skills (French or Turkish) or special qualifications (media law or web design), and (3) a biological task in which participants estimated the amount of toxin in a bug's saliva based on the bug's appearance, such as the color of the head (red or brown) or the length of the antennae (short or long). In each task the assignment of the six cues to the cue dimensions (i.e., if blood pressure denoted cue 1 or cue 2) was randomized, but the assignment of cue values as positive or negative was fixed. For instance, patients with low blood pressure always needed a higher dosage of medication than patients with a high blood pressure. For a full list of positive and negative cue values see Table 2. In addition the judgment scale changed with the three tasks. In the medical task the dosage varied between 200 and 300 mg. In the employment task the suitability was evaluated on a scale from 0 to 100 and in the biological task the toxicity of the bugs varied between 100 and 200 mg per ml. Accordingly, depending on the judgment task the criterion values reported in Table 1 were adjusted by adding 200 in the medical task and 100 in the biological bugs task.

**Table 1:** **Overview of the task structure**.

**Item no**.	**Cue 1**	**Cue 2**	**Cue 3**	**Cue 4**	**Cue 5**	**Cue 6**	**Crit**.	**Prediction: mapping strategy**	**Prediction: exemplar strategy**	**Item type**
1	0	0	0	0	1	0	1	1	1	Training
2	0	0	1	0	0	1	3	3	3	Training
3	0	1	0	0	1	1	7	9	7	Training
4	1	0	1	1	0	0	10	9	10	Training
5	1	1	1	1	0	0	27	27	27	Training
6	1	1	1	0	1	1	47	47	47	Training
7	0	0	0	1	1	1	5	9	4	*Test Q1
8	0	0	1	1	1	0	7	9	6	Test Q2
9	0	0	1	0	1	1	6	9	3	Test Q3
10	1	1	1	0	0	0	13	9	27	*Test Q1
11	1	1	0	0	0	1	10	9	27	Test Q2
12	1	1	0	1	0	0	12	9	27	Test Q3
13	1	0	0	0	0	1	4	3	3	Test
14	1	1	0	0	0	0	6	3	27	*Test
15	1	0	0	1	0	0	4	3	10	*Test
16	0	0	1	1	1	1	12	27	3	Test
17	0	1	1	1	1	0	18	27	27	*Test
18	1	0	1	1	0	1	18	27	10	*Test
19	0	1	1	1	1	1	33	47	27	Test
20	1	1	1	1	1	1	100	47	47	Test

This table indicates the cue and criterion values of the items, the predictions of the strategies and whether items appeared during training and/or test. Training items appeared during the training phase and the test phase whereas test items only appeared during the test phase. Test items with a star indicate the test items that also appeared in the fMRI test phase; Crit. stands for the items' criterion value. Cue values of “0” indicate that an item has a negative value on this cue and cue values of “1” that the item has a positive value on this cue. The test items marked with a Q denote the pairs of items with three positive cue values but opposite patterns of cue values used for the qualitative test.

**Table 2:** **Judgment scenarios**.

	**Cue 1**	**Cue 2**	**Cue 3**	**Cue 4**	**Cue 5**	**Cue 6**
**MEDICAL SCENARIO: MEDICATION DOSAGE (SCALE FROM 200 TO 300 mg)**						
Cue label	Rash	Blood pressure	Mucus	Virus	Worst period	Headache
Positive cue value	Reddening	Low	Green	Type A	Morning	In the back
Negative cue value	Itching	High	Yellow	Type B	Evening	In the front
**BIOLOGICAL SCENARIO: TOXICITY OF BUGS (SCALE FROM 100 TO 200 mg/ml)**						
Cue label	Wings	Antennae	Legs	Size	Body	Head
Positive cue value	Dotted	Long	Thick	Large	Furry	Red
Negative cue value	Uni-colored	Short	Thin	Small	Smooth	Brown
**EMPLOYMENT SCENARIO: SUITABILITY OF APPLICANT FOR IT POSITION (SCALE FROM 0 TO 100)**						
Cue label	Programming language	Foreign language	Work experience	Industrial sector	Special qualifications	Operating system
Positive cue value	Java	French	Development	Financial sector	Media law	Unix
Negative cue value	C++	Turkish	Consulting	Pharmaceutical sector	Web design	Windows

Overview of the material used. Positive cue values indicate that an item with this cue value has on average a higher criterion value than an item with a negative value on this cue.

#### Procedure behavioral judgment task

In the first session, participants were informed that they would solve two different judgment tasks using two judgment strategies. After they learnt the judgment strategy they would complete a test phase in which they had to apply both strategies. For each participant the assignment of the strategies to the task scenarios was stable throughout the study. The assignment of task scenarios to strategies and the order with which strategies were learnt was randomized between participants (i.e., one participant would learn the rule-based strategy with the medical scenario and the similarity-based strategy with the employment scenario whereas another might learn the rule-based strategy with the bugs scenario and the similarity-based strategy with the medical scenario). First, we taught participants one strategy. For this they received a description of the strategy and then completed three practice trials in which they received a detailed explanation of how to calculate the criterion value according to the strategy. After that, they practiced the strategy in a training phase. During the training phase, participants had to repeatedly judge six training items according to the strategy they had just learnt (see Table 1). In each trial participants saw one of the training items and entered their judgment. After that they received feedback on the correct criterion value of the training item and the criterion value the strategy they were learning would have estimated. Then they continued with the next trial. The training continued until they gave the same judgments as the strategy that they were instructed to use in two consecutive learning blocks with each block consisting of judging all six training items in a random order. After the first strategy had been learnt, participants rated the difficulty of the strategy on a scale from 1 (not difficult at all) to 7 (very difficult). Then the second judgment strategy was taught, practiced and the difficulty rated. Once training was completed, a test phase followed in which participants made judgments for items from both judgment strategies/tasks scenarios in a randomized order without feedback; that is, a participant that had learnt the rule-based strategy with the medical scenario and the similarity-based strategy with the employment scenario would in one trial evaluate a patient based on the patient's symptoms and in another trial the suitability of a job applicant based on the applicants' skills. In each trial, they were instructed to use the strategy with which they had learnt to solve the task during training. The test trials consisted of 40 items repeated twice, 20 from each judgment scenario. The 20 items of each task consisted of the six training items and 14 new items (see Table 1). The items for the training and test phase were selected such that the heuristic strategy and the exemplar strategy would make qualitatively different predictions. We explain these qualitative differences in detail when we report the behavioral results.

#### fMRI judgment task

The fMRI session took place at the university hospital in Basel. After arrival, participants first repeated the strategy-training phase and practiced using a button box to enter their judgments. Then they proceeded with a judgment test phase in the MRI scanner. The test phase consisted of 90 trials structured in three blocks of 30 trials. In each block participants solved 12 trials with the exemplar strategy and 12 trials with the heuristic strategy. Six of the 12 items were old items from training, six items were new (see Table 1). In the six remaining trials they worked on a distractor task in which they had to locate a spelling mistake and report the line in which it occurred. Originally the distractor task was included to allow for a comparison between the judgment task and the distractor task. However, the distractor task proved quite difficult for participants, resulting in frequent errors and making a meaningful comparison not very useful. Therefore, in our analysis we focused on direct comparison of the two judgment tasks. As a cover story for the distractor task, we used the judgment scenario out of the three scenarios described above that had not been selected for the judgment strategy training.

**Figure 1:**
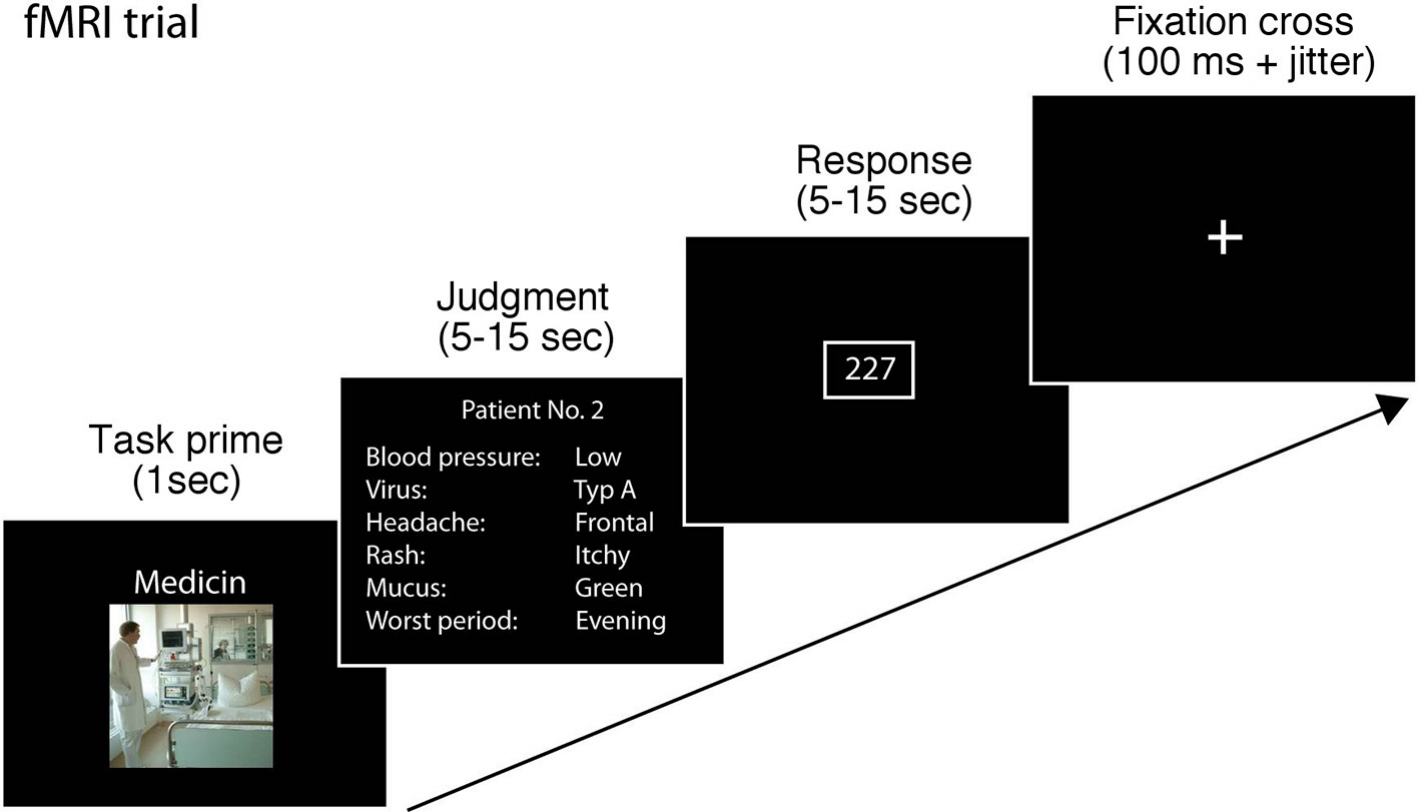
**Schematic of a single trial in the fMRI task**. A single trial consisted of fixation cross (100 ms) and a primer slide announcing the judgment scenario (1 s). After that, the information for the judgment task was provided for a minimum of 5 s and a maximum of 15 s. Participants were instructed to make their judgment and press a button as soon as they had made a decision. The next slide was presented after participants had pressed the button and 5 s passed. If 15 s passed and the participant had not pressed a button, the next slide appeared. In the next slide, participants had to enter the number they judged within 15 s by using a button box. After the trial, the fixation cross appeared and was shown for 2–4 s.

Each trial in the scanner started with a fixation cross (100 ms), followed by a primer slide announcing the judgment scenario (1 s). We included the task prime to enable participants to prepare using the correct strategy for the task and to reduce switching costs. After that, the cue information for item that had to be judged was provided for a minimum of 5 s and a maximum of 15 s. Participants were instructed to make their judgment and press a button using a button box as soon as they had made a decision. The next slide was presented after participants had pressed the button and 5 s had passed. If 15 s passed and the participant had not pressed a button, the next slide appeared. In the next slide, participants had to enter the number they judged within 15 s using the button box. To enable participants to enter the criterion value with the button box without clicking through up to 100 values, participants were taught to enter the criterion value similar to how one would set a digital clock: first they entered the value at the hundreds position, with the first button of the button box increasing the value by one and the second button decreasing the value by one. Once they had selected the desired value for the hundreds position, they could confirm it with the third button. Then they could enter the digit for the tens position confirm it and continue with the single unit position. The starting value was set at the lowest value possible in the respective task (i.e., 200 in the medical scenario, 100 in the bug task and 0 in the employment scenario). Participants received ample training with the button box before starting the session in the scanner. After the trial, the fixation cross appeared and was shown for between 2 and 4 s (see Figure 1) The session in the scanner lasted 30 min on average.

### Judgment strategies

Participants were instructed to rely on two judgment strategies, the exemplar strategy and a heuristic judgment strategy, the *mapping strategy*. To analyze whether participants indeed used the instructed strategies, we implemented computational models of the strategies and fitted them to participants' individual judgments averaged over the repeated presentations. Goodness-of-fit was measured as the root mean squared deviation (RMSD) between model predictions and participants' judgments on the new items.

#### Exemplar strategy

The exemplar strategy is a memory-based judgment strategy that assumes that judgments are made based on the similarity of the object under evaluation to previously encountered objects. Accordingly, we instructed participants to store the training objects in memory. When judging new items they were asked to retrieve the most similar item(s) from memory and make a judgment based on the criterion value of the retrieved item(s). If two items with the same similarity were retrieved, judgments should be the average of the criterion values of the two items. Participants were instructed to judge similarity between two items on the basis of their cue values; that is, the more cue values matched the higher the similarity. Accordingly, the exemplar strategy estimates, for instance, a criterion value of 27 for test item 19 (see Table 1), because it retrieves the most similar training items 3 and 6 from memory. The criterion values of these items are 7 and 47, respectively, resulting on an estimated criterion value of about 27 for item 19.

When fitting the strategy to participants' behavior we used a computational model following the implementation suggested by Juslin et al. (2008). This model follows the same basic assumptions of the exemplar strategy that we instructed participants to use but allows taking into account that people may not remember all cue dimensions. The model has been frequently and successfully used to describe human judgments when people rely on exemplar similarity (e.g., Karlsson et al., 2007; Juslin et al., 2008). This exemplar model assumes that the judgment is the average of the criterion values *x_*i*_*, of the exemplars stored in memory weighted by their similarity to the probe (i.e., the current object under evaluation).

(S1) $${\hat{y}}_{p}=\frac{\sum_{i\;=\;1}^{I}S\left(p,i\right)\;\cdot \;x_{i}}{\sum_{i\;=\;1}^{I}S\left(p,i\right)}$$

where *ŷ*_*p*_ is the estimated criterion value for the probe *p*; *S* is the similarity of the probe to the stored exemplars; *x_i_* is the criterion value of the exemplar *i*; and *I* is the number of stored exemplars in memory. The similarity *S* between the stored exemplar and the probe is calculated by the similarity rule of the generalized context model (GCM; Nosofsky, 1984):

The similarity *S*(*p*, *i*) between a probe *p* and an exemplar *i* is a nonlinearly decreasing function of their distance *d*(*p*, *i*):

(S2) $$S\left(p,\;i\right)=e^{-d(p,i)}.$$

The distance between a probe *p* and an exemplar *i* is

(S3) $$d\left(p,\;i\right)=h\left[\sum_{j\;=\;1}^{J}s_{j}\left|c_{pj}-c_{ij}\right|\right],$$

where *c_*pj*_* and *c_*ij*_*, respectively, are the cue values of the probe *p* and an exemplar *i* for the *j*th cue; *h* is a sensitivity parameter that reflects discriminability in the psychological space (e.g., Nosofsky and Zaki, 1998); and the parameters *s_j_* are the attention weights associated with cue dimension *j*. Attention weights vary between 0 and 1 and are constrained to sum to 1.

The exemplar model was fitted individually to participants' judgments of the behavioral and fMRI test phases, assuming that the six training exemplars were stored in memory and retrieved when making a judgment. Parameter values were estimated by a nonlinear, least squares optimization algorithm.

#### Mapping strategy

The mapping strategy is a rule-based heuristic strategy that allows making fast, but relatively accurate judgments (von Helversen and Rieskamp, 2008, 2009b). It makes a judgment by counting the number of positive cue values an object possesses and assigning the typical criterion value of objects with the same number of positive cues. The typical value is defined as the mean of the criterion values of objects with the same number of positive cue values. To shorten the time participants would need to learn applying this strategy, before they started with the behavioral training participants were informed which cue values in the judgment scenario in which the mapping strategy was learnt were positive. For instance, a participant learning the mapping strategy in the medical scenario would be informed in the instructions that usually patients with low blood pressure required higher doses than patients with high blood pressure and patients with green mucus required higher doses than patients with yellow mucus and so on (see Table 2). Then participants were told to count the number of positive cue values for each item they saw. For each number of positive cue values, they learnt to estimate the typical criterion value during the training phase. Accordingly, when judging test item 19, using the mapping strategy a participant would first assess the number of positive cue values (four), and then retrieve the typical criterion value for this cue sum category. The only training item with four positive cue values is item 6, which has a criterion value of 47. Thus, the typical criterion value for an item with four positive cue values is 47, which would be given as an estimate for the criterion value for the test item.

We implemented the mapping strategy as a computational model to derive its predictions (von Helversen and Rieskamp, 2008). The mapping model classified test items according to their cue sums and then estimated the typical criterion values of each cue sum category. The typical criterion values for each cue sum category were calculated based on the criterion values of the six training exemplars, or if no training exemplar with the respective cue sum was available they were based on the adjacent cue sum categories.

### Neuroimaging

Neuroimaging data were collected at the university hospital in Basel using a 3T Siemens Magnetom Verio (Erlangen, Germany) with a 12-channel head coil. Functional runs [echo-planar images (EPIs)] used a T2^*^-weighted sequence with the following acquisition parameters: repetition time (TR), 2280 ms; echo time (TE), 30 ms; flip angle (FA), 80°; field of view (FoV), 228 mm, acquisition matrix 76 × 76 with GRAPPA (generalized autocalibrating partially parallel acquisitions). Each volume consisted of 40 slices acquired parallel to the anterior commissure–posterior commissure plane (interleaved acquisition; 3 mm thick with 0 mm gap; 3 × 3 mm in-plane resolution). Stimuli were viewed through a mirror attached to the head coil and a projection screen at the back of the scanner. Each trial was associated with a different (individually jittered) EPI scan sequence. A localizer (to position the EPI), a gradient recalled echo (GRE) field map, and a T1 (TR, 2000 ms; TE, 3.37 ms; FA, 8°; FoV, 256, 176 sagittal slices, three-dimensional acquisition; 1 mm thick; 1 × 1 mm in-plane resolution; 256 × 256 matrix) were run before the EPI scan.

Preprocessing was performed using SPM8 (Statistical Parametric Mapping, Wellcome Department of Cognitive Neurology, London, UK) implemented in Matlab 2010Ra (Mathworks Inc., Natick, MA, USA). The first three volumes of each run were discarded to allow for magnetic saturation effects. Volumes were slice-time corrected to the first slice and realigned to the first acquired volume. A mean image created from the realigned volumes was co-registered with the structural T1 volume and the structural volumes were spatially normalized. The spatial transformation was applied to the realigned T2 volumes, which were spatially smoothed using a Gaussian kernel of 8 mm full-width half maximum.

#### General linear model analyses

For the statistical evaluation, we defined a general linear model that included four regressors of interest and 10 regressors of non-interest. The four regressors of interest consisted of the judgment items for new and old items for the exemplar task and the mapping task (“Exemplar Task New Items,” “Exemplar Task Old Items,” “Mapping Task New Items,” “Mapping Task Old Items”). Onsets for these regressors were at the time when the judgment items appeared on screen. Participants indicated when they had formed a judgment by a button press. We used the response times to model the duration of each event.

The regressors of no interest consisted of the distractor task, the task prime (1 s), the response screen (duration modeled with participants' response times), and the fixation cross (2–4 s). All regressors were convolved with a canonical hemodynamic response function. Last, the six scan-to-scan motion parameters produced during realignment were included as additional parametric regressors in the SPM analysis to account for residual effects of scan-to-scan motion.

We computed linear contrasts of regression coefficients at the individual subject level and then took them to a second-level random effects analysis. We calculated the following contrast images: *Mapping Task New Items > Exemplar Task New Items*, *Exemplar Task New Items > Mapping Task New Items, Mapping Task Old Items > Exemplar Task Old Items* and *Exemplar Task Old Items > Mapping Task Old Items*. Because judgments for the old items not only depend on the strategies, but also involve other processes such as recognition memory, the first two contrasts are the most diagnostic for comparing the two strategies and will thus be the main focus of the results and the discussion. For second-level random effects analysis, the single-subject contrasts were entered into one-sample *t*-tests. All statistical images were thresholded with *p* < 0.0001 uncorrected and a cluster significance level of *p* < 0.05, corrected for multiple comparisons at the cluster level (FWE), for clusters with more than 20 voxels (see Thirion et al., 2007; Gureckis et al., 2011 for a similar procedure). Activations were located based on the Automated Anatomical Labeling atlas and manually checked (AAL; Tzourio-Mazoyer et al., 2002). Locations of peak activations are indicated in MNI (Montreal Neurological Institute) space. To determine Brodmann areas, we converted the MNI coordinates of the peak activations into Talairach space (Lancaster et al., 2007) and then used the Talairach daemon to locate the closest Brodmann area (Lancaster et al., 1997, 2000).

## Results

### Behavioral data

**Figure 2:**
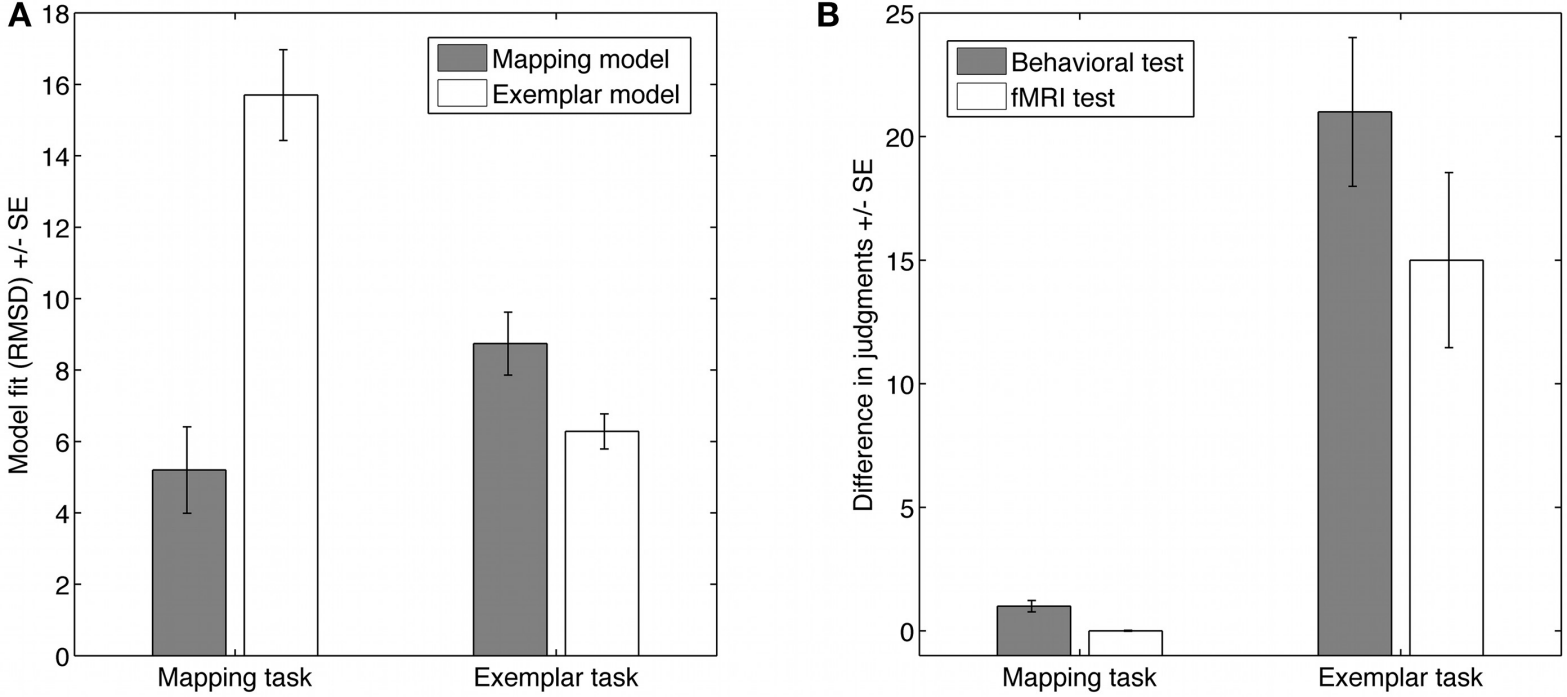
**Behavioral evidence for the two strategies in the two tasks. (A)** Shows the model fits for participants' judgments of the new items in the two tasks. **(B)** Shows the differences in judgments for item pairs with always three positive cue values each, but different cue values on each cue dimension. The results for the behavioral and the imaging study show that, as predicted by the mapping model, participants made similar judgments for these item pairs in the mapping task, but as predicted by the exemplar strategy made different judgments in the exemplar task (error bars = 1 SE).

We analyzed the behavioral judgments in the behavioral test phase and the fMRI test phase together to increase the reliability of the strategy assessment. On average participants made similar judgments in both tasks. Using a scale from 0 to 100, mean judgments were *M* = 14.2 (*SD* = 13.8) in the mapping task and *M* = 14.6 (*SD* = 8.4) in the exemplar task. Also, the spread of judgments was comparable, ranging from 1 to 47 during the fMRI test phase for both tasks. Thus, on the average behavioral level participants appear to solve the task in a similar way. However, having a closer look confirmed that the judgments were generated by different strategies. Focusing on the more diagnostic new items, Figure 2 (left panel) illustrates that for the mapping task, the mapping model described on average participants' judgments better than the exemplar model, whereas for the exemplar task the exemplar model described on average participants' judgments better than the mapping model. This was supported by a repeated measurement analysis of variance with model and task as within-subject factors resulting in a highly significant interaction, [*F*_(1, 17)_ = 79.9, *p* < 0.001, partial η^2^ = 0.83]^1^. Nevertheless, in the exemplar task the mapping model fitted the judgments of two participants better than the exemplar model. Thus, we excluded these two participants from further analyses.

Although judgments are comparable on the mean level, an analysis of the judgments on the item level for the new items showed that participants' judgments differed in the two conditions as predicted. For a strong qualitative test of the models we had selected the new items so that the strategies make opposing judgments for pairs of items (for this qualitative test see also von Helversen and Rieskamp, 2009a). Specifically we created pairs of items for which each item always had three positive cues values out of the six cues; however, which cue had a positive value was different for all cues between the pairs of items. For these specific pairs of items the mapping model predicts the same value for the two items with three positive cue values even though these two items had no single dimension out of six with the same cue values. In contrast, these two items were treated quite differently by the exemplar model, which made very different predictions (see also Table 1). Figure 2 (right panel) illustrates that the judgments were in line with the predictions, so that participants made similar judgments for the item pairs in the mapping task but made different judgments in the exemplar task. Additionally we analyzed how easily participants learnt the strategies, how difficult they judged using a strategy, and how long they took to give a judgment using the strategies. Overall, the results suggest that the exemplar strategy was more difficult than the mapping strategy. Participants required more training trials to learn the exemplar strategy than the mapping strategy (*M*_Exemplar_ = 96, *SD* = 52; *M*_Mapping_ = 33, *SD* = 21), they reported it as more difficult to use after learning it [*M*_Exemplar_ = 4.7, *SD* = 1.7; *M*_Mapping_ = 2.9, *SD* = 1.2; *t*_(17)_ = 4.63, *p* < 0.001], and were slower in the exemplar task than in the mapping task in the fMRI test phase [*M*_Exemplar = 6.9 s_, *SD* = 2.2; *M*_Mapping_ = 5.5 s, *SD* = 1.3; *t*_(17)_ = 2.71, *p* < 0.02].

### Neuroimaging

**Figure 3:**
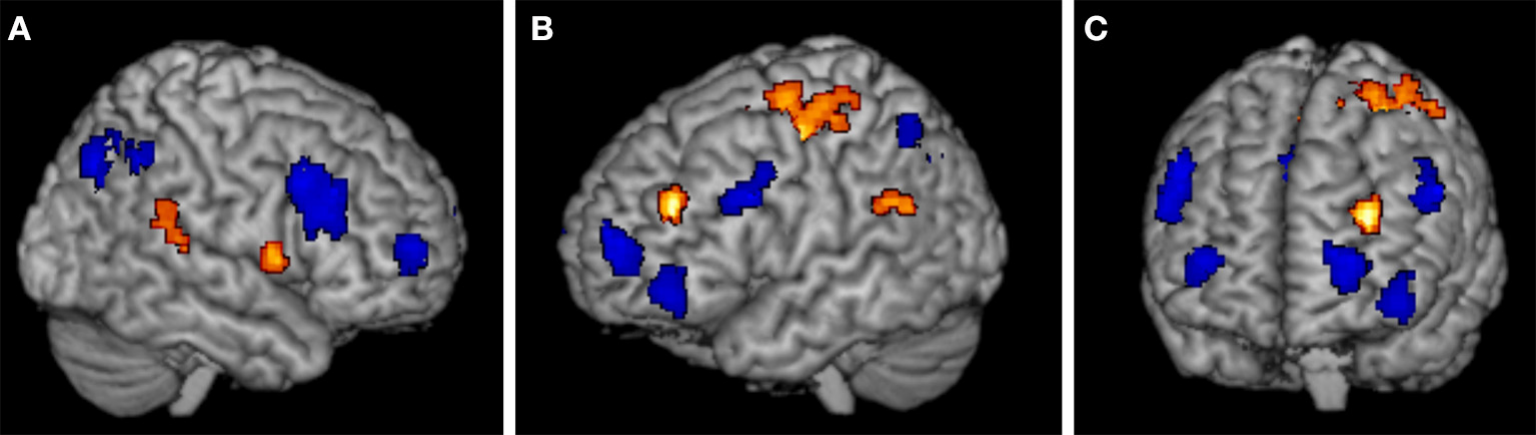
**Regions with significant activation differences in (A) the right cortex, (B) the left cortex, and (C) frontal cortex, with threshold *p* < 0.0001 (cluster level: *p* < 0.05, FWE)**. Regions that were more highly activated in the exemplar task are shown in blue, regions that were more highly activated in the mapping task are in orange.

To compare brain activation when using the exemplar strategy to when using the mapping strategy, we calculated contrasts between the trials of the mapping task and the exemplar task involving old (training) items and new items, where the comparison of new items is more informative. The results for the new items show that the strategies rely partially on different neural substrates involving prefrontal and parietal regions. As shown in Figures 3A–C, in the exemplar task we found higher activations relative to the mapping task bilaterally in the (aPFC) at the superior frontal gyrus (−27, 62, 13; 33, 59, 10) and the left inferior frontal gyrus (−42, 47, −8). In addition we found higher activations in the dlPFC and the ventrolateral prefrontal cortex (vlPFC) at the inferior frontal gyrus (48, 17, 37) on the right side and between the dlPFC and the vlPFC at the inferior frontal sulcus (−54, 17, 34) on the left side. Furthermore, we found higher activations in the IPC on the right at the inferior parietal lobe (36, −67, 37) and on the left at the angular gyrus (−36, −58, 43). For a full list of activations, see Table 3. In contrast, in the mapping task relative to the exemplar task, we found higher activations in the prefrontal cortex in the left dlPFC at the middle frontal gyrus (−30, 41, 25) and the right vlPFC at the inferior frontal gyrus (57, 8, 7), see Figure 3A. In the parietal cortex we found higher activations in the left temporal-parietal cortex at the supramarginal gyrus (−57, −43, 28). In addition, we found higher activations in the precentral and postcentral gyrus and supplementary motor areas. See Table 3 for a full list of activations.

**Table 3:** **Activation differences between the exemplar and the mapping task for new items**.

**Region**	**Hem**.	**BA**	**MNI coordinates**			**No. of voxels**	***Z***	***P***
			***x***	***y***	***z***			
**EXEMPLAR TASK > MAPPING TASK NEW ITEMS**								
Inferior frontal sulcus	L	9	−54	17	34	51	4.97	0.001
Inferior parietal lobe	R	19	36	−67	37	151	4.88	<0.001
Inferior frontal gyrus	R	9	48	17	37	155	4.76	<0.001
Superior frontal gyrus	L	10	−27	62	13	53	4.72	0.001
Medial superior frontal gyrus	L	32	−3	29	43	63	4.61	<0.001
Cerebellum Crus2	L		−9	−79	−29	32	4.56	0.006
Superior frontal gyrus	R	10	33	59	10	42	4.54	0.002
Inferior frontal gyrus	L	10	−42	47	−8	47	4.40	0.001
Inferior parietal lobe Angular gyrus	L	39	−36	−58	43	57	4.27	<0.001
**MAPPING TASK > EXEMPLAR TASK NEW ITEMS**								
Precentral gyrus	L	4	−27	−13	52	155	5.07	<0.001
Middle frontal gyrus	L	9	−30	41	25	42	4.92	0.002
Supplementary motor area	R	6	12	−4	52	217	4.77	0.001
Inferior frontal gyrus	R	44	57	8	7	23	4.37	0.019
*Supramarginal gyrus	L	40	−57	−43	28	21	4.20	0.024
*Postcentral gyrus	L	40	−33	−34	49	25	4.18	0.014
Superior temporal gyrus	R	42	69	−28	13	23	3.99	0.019

N = 18. Hem, hemisphere; R, right; L, left; BA, Brodmann area; Z, z-value for peak voxel, number of voxels indicated as thresholded at p < 0.0001, uncorrected; P, p-value for the cluster, corrected for multiple comparisons at the cluster level (p < 0.05, FWE). Areas that were no longer significant in analyses controlling for differences in subjective difficulty and response times are marked with a ^*^.

The behavioral results had shown differences in the perceived difficulty of the strategies and response times. Thus, to ensure that the differences in neural activation were not mainly caused by differences in strategy difficulty, we ran further second-level analyses statistically controlling for difference in mean response times and difference in perceived strategy difficulty. To do this we first calculated for each participant the difference in difficulty ratings between the two strategies and the difference in mean response times for the trials in which the mapping and the exemplar strategy had been used. Then we added both variables as additional covariates in the second-level analysis using the same statistical thresholds as in the original analysis (*p* < 0.0001, *k* = 20). The analysis showed the same pattern of activations for the exemplar task > mapping task contrast. For the mapping task > exemplar task contrast we also found a similar pattern of results, but the activation in the left parietal lobe was somewhat reduced (see Table 3). In sum, these results suggest that the differences between the two types of strategies cannot simply be explained by strategy difficulty.

The results for the old items also show a similar but somewhat weaker pattern of results than the new items (see Table 4) suggesting that, overall, participants used the instructed strategy for old and new items. The results for the old items, however, are less diagnostic regarding the neural processes underlying the strategies than the new items, because the old items had been extensively practiced previously and thus participants could have recruited additional judgment processes such as recognition memory.

**Table 4:** **Activation differences between the exemplar and the mapping task for old items**.

**Region**	**Hem**.	**BA**	**MNI coordinates**			**No. of voxels**	***Z***	***P***
			***x***	***y***	***z***			
**EXEMPLAR TASK > MAPPING TASK OLD ITEMS**								
Inferior frontal gyrus	L	46	−42	44	−5	94	4.79	<0.001
Medial superior frontal gyrus	L	8	−3	26	43	44	4.61	0.001
Middle frontal gyrus	L	8	−51	17	37	36	4.55	0.002
**MAPPING TASK > EXEMPLAR TASK OLD ITEMS**								
Superior temporal gyrus	L	42	−57	−31	19	259	5.49	<0.001
Postcentral gyrus	R	7	9	−37	55	997	5.39	<0.001
Superior temporal gyrus	R	13	45	−40	19	398	5.26	<0.001
Rolandic operculum	L	22	−57	5	1	99	4.55	<0.001
Putamen	L		−30	−13	10	33	4.38	0.003
Middle cingulate gyrus	R	24	6	11	34	20	4.30	0.018
Postcentral gyrus	L	4	−54	−7	40	27	4.19	0.006

N = 18. Hem, hemisphere; R, right; L, left; BA, Broadman area; Z, z-value for peak activation, number of voxels indicated as thresholded at p < 0.0001, uncorrected; P, p-value for the cluster, corrected for multiple comparisons at the cluster level.

## Discussion

The neural substrates of judgments have sparked considerable interest (e.g., Greene and Haidt, 2002; Paulus and Frank, 2003; Heekeren et al., 2005; Knutson et al., 2007; Park et al., 2011). Relatively little attention has been given to the intra-individual variability in strategies when people make judgments. Behavioral research has illustrated people's flexibility in solving judgment problems: people often apply different strategies, even for solving identical problems (Juslin et al., 2008). This variability must to some extent be reflected in variability in the neural substrates underlying judgments. Without a method to capture the strategy used, such as cognitive modeling, the neural activation can easily be misinterpreted. For instance, investigating neural activations in judgments under cognitive load—a situation that sometimes induces strategy shifts (Hoffmann et al., 2013)—the results should differ widely, depending on whether a strategy shift occurred or not. In contrast, taking into account the strategies people use can inform imaging research by guiding predictions about the neural substrates involved. For instance, the activation of areas involved in memory retrieval appears to be essential from the perspective of an exemplar strategy, but not when a rule-based strategy is employed. Furthermore, if unique neural signatures for strategies could be identified, it could make it possible to deduce the strategy a person is using from the neural activations.

In the present work we took a first step, aiming to show that two judgment strategies have different neural signatures in the identical judgment problem and to compare them to research investigating the differences between similarity-based and rule-based strategies in categorization. Overall, our results show activations in areas that have been frequently implicated in research on the neural substrates of complex judgments such as the ventromedial prefrontal cortex, the dlPFC and the PPC (Greene and Haidt, 2002; Paulus and Frank, 2003; Kahnt et al., 2011) but also in categorization (Grossman et al., 2002; Koenig et al., 2005; Li et al., 2007).

In the following, we will discuss how the findings relate to the cognitive processes that the strategies assume and the categorization literature.

### Similarity-based judgments: exemplar strategy

Consistent with our hypotheses and resonating well with research on memory retrieval, we found that when using the exemplar strategy participants had higher bilateral activation in the aPFC and the IPC than when using the mapping strategy. The aPFC is commonly recruited in the retention and retrieval of semantic material from long-term memory (Buckner and Koutstaal, 1998; Lepage et al., 2000; De Zubicaray et al., 2001). Meta-analyses on retrieval processes in memory have identified comparable parietal and frontal areas (Spaniol et al., 2009; Kim, 2011). We did not find increased activation in the medial temporal lobe or the hippocampus. Yet, the hippocampus has not consistently been reported in memory retrieval (see Henson, 2005). Also, a meta-analysis suggested that the medial temporal areas are more strongly involved in encoding than in retrieval (Spaniol et al., 2009).

Additionally, we found a higher activation for the exemplar strategy in the dlPFC, reaching into the mid vlPFC on the right side and between the dlPFC and the vlPFC on the left side. Although activation in the dlPFC is often linked to executive functioning and thus may be expected when using a rule-based strategy such as the mapping strategy (e.g., Grossman et al., 2002; Filoteo et al., 2005), it is frequently implicated in memory processes. For instance, the right dlPFC has been connected to spatial working memory (McCarthy et al., 1994) and the vlPFC to the cognitive control of memory (Badre and Wagner, 2007; Martin, 2007). Specifically, the mid vlPFC is involved in the selection of competing traces in memory retrieval (Badre et al., 2005; Spaniol et al., 2009).

Our results on similarity-based judgments also resonate with studies comparing similarity- and rule-based strategies in categorization. For instance, Koenig et al. (2005) also reported higher activation for similarity-based vs. rule-based strategies in the aPFC. However, studies comparing rule- with similarity-based categorization have usually reported higher activation in the parietal and occipital cortex for rule-based strategies than for similarity-based strategies (Patalano et al., 2001; Filoteo et al., 2005; Koenig et al., 2005; Seger and Miller, 2010). In contrast, we found a higher activation for the exemplar strategy than the mapping strategy in the parietal cortex. Although these results seem to contradict our results at first glance, they can be reconciled when considering the differences between rule-based and similarity-based strategies in categorization and judgment. Activation in the IPC and parietal-occipital lobe is often linked to perceptual processing and selective attention (Wager and Smith, 2003; Smith and Grossman, 2008). The rule-based strategies investigated in categorization usually require focusing attention on a single cue dimension such as deciding whether a bug is toxic solely based on the color of its head. In contrast, similarity-based strategies usually involve attention to several or all dimensions such as considering a bug as toxic if it overall resembles toxic bugs, thus considering, for instance, not only the color of the head but also the size or whether the wings are dotted. In contrast, in our tasks the exemplar strategy allowed more selective attention to specific cue dimension than the mapping strategy. Even though we instructed participants to consider all cues, this is difficult to do and the strategy may become easier to implement if not all, but only some of the dimensions are considered. For instance, a participant following a similarity-based strategy could have determined the similarity simply by focusing on three of the six cues when retrieving the training exemplars. In contrast, the mapping strategy requires equal attention to all cues, because all positive cue values need to be counted. Thus, to the degree that the areas are involved in the perceptual processing of increased attentional demands (Wager and Smith, 2003; Smith and Grossman, 2008), a higher activation when using the exemplar strategy can be expected in our comparison. This interpretation is also supported by research showing that single-dimensional sorting compared to multi-dimensional sorting (i.e., categorization without feedback) induced higher activation in the parietal and temporal lobe (Milton et al., 2009).

### Rule-based judgments: mapping strategy

The mapping strategy is an explicit, rule-based strategy involving the classification of an object to a category based on the number of positive cue values and the selection of a judgment response depending on the classification. These cognitive processes draw upon cognitive control functions. This, in turn, suggests the involvement of the lateral prefrontal cortex (e.g., Wagner et al., 2001; Wager and Smith, 2003). When the mapping strategy was employed we consistently found higher activations relative to the exemplar strategy in the left dlPFC and the right vlPFC. Both areas are often involved in cognitive tasks that require control resources and have been related to the processing demands involved in the explicit application of rules (Reber et al., 1998; Elliott et al., 1999; Patalano et al., 2001; Savage et al., 2001; Wallis and Miller, 2003; Filoteo et al., 2005; Zysset et al., 2006; Smith and Grossman, 2008). In addition, the dlPFC has been implicated in multi-attributive decision-making (Zysset et al., 2006; Li et al., 2007; Khader et al., 2011). The results also resonate with differences in dlPFC activity found in the comparison between semantic rule-based categorization and similarity-based categorization (e.g., Grossman et al., 2002). Thus, our finding of activation in the dlPFC for both rule-based and similarity-based strategies could suggest that depending on the type of strategy used different regions of the dlPFC are involved. Possibly, memory-based strategies such as the exemplar model could involve the ventral dlPFC close to the vlPFC, whereas rule-based strategies recruit areas in the mid dlPFC.

In contrast to rule-based strategies in research on categorization, the mapping strategy requires integrating information by grouping relatively unrelated information into a common category. For instance, in the cue sum category with three positive values, items that do not have a single cue value in common are grouped together. Consistent with this assumption and contrary to studies comparing rule-based to similarity-based strategies in categorization (e.g., Grossman et al., 2002), we found higher activation in the mapping task than in the exemplar task in the temporal-parietal cortex. This area has been implicated in intuitive judgment processes (Ilg et al., 2007), and seems to be involved in holistic processing and the integration of distantly related semantic information into a coherent representation (Beauchamp et al., 2004; Jung-Beeman et al., 2004). This suggests that differences to the categorization literature are caused by the differences in the type of rule-based strategies considered in these areas of research, specifically the number of dimensions that need to be integrated.

In addition, we found relatively higher activations in primary and supplementary motor areas, which could result from activation related to planning motor action, but have also been connected to decision making and rule use (e.g., Wallis and Miller, 2003; Cisek, 2006; Klaes et al., 2011).

One limitation of this study is that we focused on the execution of instructed strategies. This approach was necessary to ensure that participants rely on the suggested strategies but good evidence exists that these strategies are used when making decisions spontaneously in similar tasks (Karlsson et al., 2008; Hoffmann et al., 2013). Furthermore, our study involved extensive training, suggesting that participants did not have to intentionally recall which strategy to use. Nevertheless, strategies such as the exemplar strategy that rely on associative and implicit processes may involve different patterns when spontaneously and unintentionally used. Future research is necessary to investigate whether similar pattern of activations are found when participants spontaneously employ different judgment strategies. Furthermore, it would be interesting to investigate whether similar brain areas are involved when different type of rule-based strategies are instructed or spontaneously employed.

In addition, it should be noted that our results are based on artificial laboratory tasks. This was necessary to enable participants to learn the judgment task and to control for prior knowledge. However, cognitive processes as well as brain areas involved in laboratory tasks could differ from real-world judgment tasks.

## Conclusion

We investigated the neural patterns underlying human judgment. We illustrate that the identical problem can be solved with quite different strategies. The results show that the two judgment strategies we examined—strategies that have frequently been reported as successful models predicting human judgment—involve different neural correlates. Thus, the study illustrates that the two judgment strategies have specific cognitive demands that are reflected in the pattern of neural activations. The findings highlight how neuroimaging can be used to better understand the cognitive mechanisms involved in judgment and decision making. Furthermore, the findings emphasize the importance of taking different strategies into account when interpreting neural activation in cognitive tasks, as the same task can involve different neural correlates depending on the strategy employed.

### Conflict of interest statement

The authors declare that the research was conducted in the absence of any commercial or financial relationships that could be construed as a potential conflict of interest.

## Footnotes

^1^The same conclusion holds when using the Bayesian information criterion (BIC; Schwartz, 1978; Raftery, 1995) as measure of model fit, which takes the models' complexities (i.e., their number of free parameters) into account.
